# Supplementary material for: Hot spot prediction in protein-protein interactions by an ensemble system
Source: BMC Syst Biol. 2018 Dec 31;12(Suppl 9):132. doi: 10.1186/s12918-018-0665-8 (PMC6311905; doi:10.1186/s12918-018-0665-8)
Supplement: Supplementary file 2 — All relevant MATLAB code. A simple Matlab implement of our predictor. (ZIP 19,742 kb) [file 12918_2018_665_MOESM2_ESM.zip › Additional file 2/Read ASA/ASA Property/SKEMPI/3SGBI.txt]

 NetSurfP - Protein Surface Accessibility and Secondary Structure Predictions

  

|  |  |  |
| --- | --- | --- |
|  |  | NetSurfP - Protein Surface Accessibility and Secondary Structure Predictions  Technical University of Denmark |

  


---

```
# For publication of results, please cite:
# A generic method for assignment of reliability scores applied to solvent accessibility predictions.
# Bent Petersen, Thomas Nordahl Petersen, Pernille Andersen, Morten Nielsen and Claus Lundegaard
# BMC Structural Biology 2009, 9:51 doi:10.1186/1472-6807-9-51
#
# Column 1: Class assignment - B for buried or E for Exposed - Threshold: 25% exposure, but not based on RSA
# Column 2: Amino acid
# Column 3: Sequence name
# Column 4: Amino acid number
# Column 5: Relative Surface Accessibility - RSA
# Column 6: Absolute Surface Accessibility
# Column 7: Z-fit score for RSA prediction
# Column 8: Probability for Alpha-Helix
# Column 9: Probability for Beta-strand
# Column 10: Probability for Coil
E D  3SGBI                  1    0.915 131.880   1.074   0.003   0.003   0.994
B C  3SGBI                  2    0.222  31.197  -2.654   0.018   0.019   0.964
E S  3SGBI                  3    0.693  81.231   0.198   0.058   0.017   0.925
E E  3SGBI                  4    0.652 113.817   0.755   0.058   0.017   0.925
E Y  3SGBI                  5    0.299  63.832  -0.038   0.058   0.017   0.925
E P  3SGBI                  6    0.572  81.153   0.525   0.053   0.043   0.903
E K  3SGBI                  7    0.581 119.429   0.183   0.052   0.084   0.864
E P  3SGBI                  8    0.327  46.415  -0.006   0.052   0.084   0.864
E A  3SGBI                  9    0.309  34.008  -0.416   0.052   0.084   0.864
B C  3SGBI                 10    0.049   6.837   0.002   0.018   0.047   0.935
E T  3SGBI                 11    0.341  47.255  -0.416   0.053   0.043   0.903
E L  3SGBI                 12    0.420  76.847  -0.583   0.052   0.084   0.864
E E  3SGBI                 13    0.434  75.802  -0.511   0.056   0.142   0.802
B Y  3SGBI                 14    0.200  42.719   0.068   0.056   0.142   0.802
E R  3SGBI                 15    0.400  91.646  -0.392   0.052   0.084   0.864
B P  3SGBI                 16    0.115  16.304  -0.825   0.052   0.084   0.864
B L  3SGBI                 17    0.098  17.871   0.151   0.064   0.216   0.721
B C  3SGBI                 18    0.056   7.834   0.258   0.064   0.216   0.721
B G  3SGBI                 19    0.166  13.025  -1.203   0.056   0.142   0.802
E S  3SGBI                 20    0.398  46.646  -1.003   0.053   0.043   0.903
E D  3SGBI                 21    0.488  70.306  -0.578   0.018   0.019   0.964
E N  3SGBI                 22    0.383  56.130  -1.599   0.018   0.019   0.964
E K  3SGBI                 23    0.381  78.269   0.304   0.018   0.088   0.893
B T  3SGBI                 24    0.274  38.018  -0.533   0.020   0.205   0.775
B Y  3SGBI                 25    0.153  32.760   0.706   0.118   0.150   0.732
E G  3SGBI                 26    0.506  39.814  -0.902   0.257   0.016   0.727
B N  3SGBI                 27    0.242  35.400  -1.168   0.430   0.016   0.555
E K  3SGBI                 28    0.372  76.562   0.626   0.751   0.050   0.199
B C  3SGBI                 29    0.044   6.107   0.461   0.779   0.100   0.120
E N  3SGBI                 30    0.308  45.062   0.863   0.779   0.100   0.120
B F  3SGBI                 31    0.093  18.745   0.699   0.870   0.077   0.053
B C  3SGBI                 32    0.080  11.204  -0.404   0.870   0.077   0.053
B N  3SGBI                 33    0.256  37.434   0.167   0.870   0.077   0.053
E A  3SGBI                 34    0.303  33.391  -0.237   0.779   0.100   0.120
B V  3SGBI                 35    0.259  39.747   0.337   0.746   0.197   0.057
B V  3SGBI                 36    0.162  24.961   0.476   0.662   0.225   0.113
E E  3SGBI                 37    0.539  94.093   0.181   0.428   0.171   0.402
E S  3SGBI                 38    0.542  63.558  -1.039   0.113   0.087   0.800
E N  3SGBI                 39    0.669  97.912  -0.306   0.018   0.019   0.964
E G  3SGBI                 40    0.303  23.822  -0.724   0.018   0.047   0.935
E T  3SGBI                 41    0.479  66.410   1.241   0.005   0.336   0.660
B L  3SGBI                 42    0.043   7.800   0.368   0.002   0.816   0.182
E T  3SGBI                 43    0.313  43.482   0.962   0.011   0.918   0.071
B L  3SGBI                 44    0.100  18.365   0.801   0.011   0.918   0.071
E S  3SGBI                 45    0.374  43.880   1.037   0.011   0.918   0.071
B H  3SGBI                 46    0.279  50.786  -0.339   0.018   0.846   0.136
E F  3SGBI                 47    0.497  99.668  -0.351   0.004   0.514   0.481
E G  3SGBI                 48    0.295  23.224  -2.686   0.005   0.262   0.733
E K  3SGBI                 49    0.652 134.219   1.176   0.004   0.197   0.799
E C  3SGBI                 50    0.525  73.738  -2.042   0.003   0.003   0.994

Explain the output.  Go back.
```

---
